# Antiphospholipid antibodies enhance rat neonatal cardiomyocyte apoptosis in an *in vitro* hypoxia/reoxygenation injury model via p38 MAPK

**DOI:** 10.1038/cddis.2016.235

**Published:** 2017-01-12

**Authors:** Lauren T Bourke, Thomas McDonnell, James McCormick, Charis Pericleous, Vera M Ripoll, Ian Giles, Anisur Rahman, Anastasis Stephanou, Yiannis Ioannou

**Affiliations:** 1 Centre for Rheumatology, Division of Medicine University College London, Rayne Institute, London, UK; 2 Arthritis Research UK Centre for Adolescent Rheumatology, University College London, London, UK; 3 Biochemistry Research Group, Clinical and Molecular Genetics Unit, Institute of Child Health & Great Ormond Street Hospital, University College London, London, UK; 4 Medical and Molecular Biology Unit, University College London, London, UK

## Abstract

A significant amount of myocardial damage during a myocardial infarction (MI) occurs during the reperfusion stage, termed ischaemia/reperfusion (I/R) injury, and accounts for up to 50% of total infarcted tissue post-MI. During the reperfusion phase, a complex interplay of multiple pathways and mechanisms is activated, which ultimately leads to cell death, primarily through apoptosis. There is some evidence from a lupus mouse model that lupus IgG, specifically the antiphospholipid (aPL) antibody subset, is pathogenic in mesenteric I/R injury. Furthermore, it has previously been shown that the immunodominant epitope for the majority of circulating pathogenic aPLs resides in the N-terminal domain I (DI) of beta-2 glycoprotein I (*β*_2_GPI). This study describes the enhanced pathogenic effect of purified IgG derived from patients with lupus and/or the antiphospholipid syndrome in a cardiomyocyte H/R *in vitro* model. Furthermore, we have demonstrated a pathogenic role for aPL containing samples, mediated via aPL–*β*_2_GPI interactions, resulting in activation of the pro-apoptotic p38 MAPK pathway. This was shown to be inhibited using a recombinant human peptide of domain I of *β*_2_GPI in the fluid phase, suggesting that the pathogenic anti-*β*_2_GPI antibodies in this *in vitro* model target this domain.

The antiphospholipid syndrome (APS) is characterised by the clinical syndrome of arterial and venous thrombosis and/or recurrent pregnancy morbidity in association with the presence of antiphospholipid (aPL) antibodies.^1^ Beta-2 glycoprotein I (*β*_2_GPI) consists of five homologous peptide subunits, which are termed domains I to V^2, 3^ with domain V being responsible for binding anionic phospholipid.^4^ A subpopulation of aPL derived from patients with APS binds phospholipids via domain I of the protein co-factor *β*_2_GPI to promote thrombosis,^5, 6^ and these anti-DI antibodies have been proven to be pathogenic in an animal model of venous thrombosis.^7, 8^

Myocardial infarction (MI) is an important cause of morbidity and mortality in systemic lupus erythematosus (SLE) and many patients with SLE also have co-existing APS. It is widely accepted that accelerated atherosclerosis is a key contributor to cardiovascular-related morbidity and mortality in lupus; however, the role of I/R injury is yet to be explored. APS is associated with a greater risk of atherosclerosis compared with matched controls^9^ and accounts for one in nine MIs.^10^ There is some evidence within a lupus mouse mesenteric I/R injury model that lupus IgG are pathogenic.^11^ Additionally aPL, which are found in 30–40% of lupus patients,^12^ as well as those with only APS (primary APS) has been shown to specifically restore mesenteric I/R injury in a complement deficient mouse model.^13^ Therefore, if relevant to the myocardium, one might expect that cardiac infarct size post-MI would be larger in patients with SLE and/or APS. However, to our knowledge no large-scale study has been undertaken to compare cardiac infarct sizes between patients with SLE±APS and age- and gender-matched controls. Given that patients with SLE have greater cardiovascular risks and increased mortality due to cardiovascular events,^14^ it is relevant to investigate if, at the experimental level, IgG from SLE and/or APS enhance cardiac I/R injury.

During the reperfusion phase of I/R injury, a complex interplay of multiple pathways and mechanisms is activated, which ultimately culminate in cell death primarily through apoptosis. *In vivo* rat studies have shown that the number of TUNEL (terminal deoxynucleotidyl transferase-mediated dUTP nick-end labelling)-positive cardiomyocytes in I/R injury can be reduced by treatment with a caspase inhibitor,^15^ resulting in a reduced infarct size indicating that apoptosis plays an important role in I/R-induced damage. The mitogen-activated protein kinase (MAPK) family, which consists of a series of serine–threonine kinases, is known to play a role in cell proliferation, differentiation and survival, but have also been shown to be heavily implicated in I/R injury in both a pro- and anti-apoptotic fashion. Specifically, *in vivo* animal studies have shown that inhibition of p38 MAPK via SB203580 results in a reduction in myocardial damage^16^ and improved cardiac function.^17^ Inhibition of p38 MAPK also leads to a reduction in inflammatory cytokines such as tumour necrosis factor alpha (TNF-*α*), interleukin-1 (IL-1) and interleukin-8 (IL-8), therefore reducing the pro-inflammatory response which is known to contribute to cardiac I/R injury.^18^

In the presence of serum co-factors, such as *β*_2_GPI, pathogenic anti-PL binds negatively charged PL such as cardiolipin (CL).^19^ This has been shown to result in an aPL-mediated activation of p38 MAPK and NF-*κ*B pathways in cultured human endothelial cells^20^ and monocytes.^21, 22, 23^ One may hypothesise that if *β*_2_GPI-dependent aPL were to also activate p38 MAPK in cardiomyocytes, then this would enhance apoptosis in cardiac I/R injury.

Here, we describe a pathogenic role for aPL antibodies in an *in vitro* model of cardiomyocyte hypoxia/reoxygenation (H/R) injury. This injury is shown to be induced via the pro-apoptotic p38 MAPK. Furthermore, in the absence of *β*_2_GPI or the presence of a recombinant human DI decoy peptide pathogenicity was abrogated, suggesting this effect is mediated through antibodies targeting DI of *β*_2_GPI.

## Results

### Clinical and laboratory characteristics of individuals

A total of 11 patients with APS, 9 SLE/aPL negative (APS negative) and 15 healthy controls (HCs) subjects were studied (Table 1). Of the 35 subjects, 28 were female. Of 11 patients with APS, 5 had SLE (clinical and serological details are shown in Table 1) and 6 primary APS.

### IgG purified from patients with SLE enhances apoptosis in neonatal rat cardiomyocytes within an *in vitro* H/R injury model

**Figure 1:**
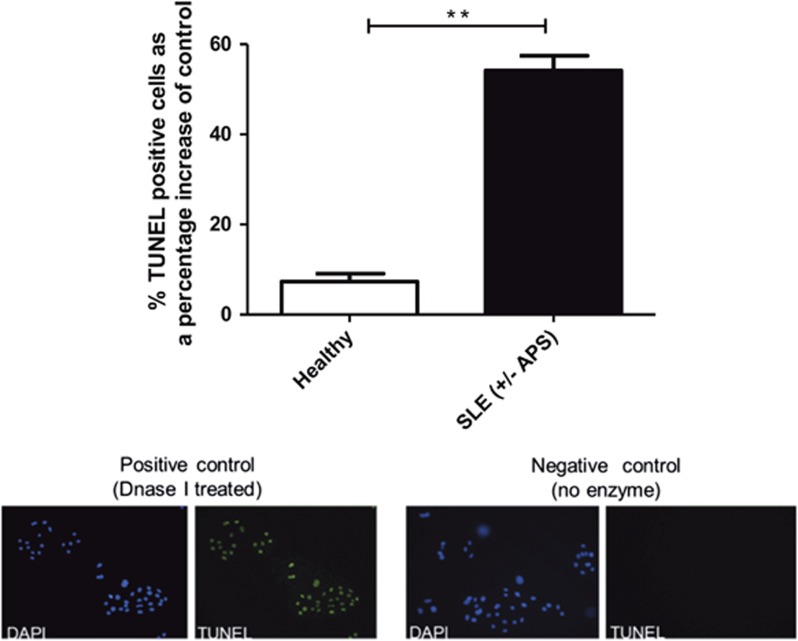
IgG from patients with SLE enhances the number of TUNEL-positive cells in neonatal rat cardiomyocytes exposed to H/R injury. Neonatal rat cardiomyocytes were pre-treated with 500 *μ*g/ml IgG from either healthy or SLE patients and the following day cells were exposed to H/R injury (4 h hypoxia+16 h reoxygenation). Cells were fixed in 4% PFA and the percentage of TUNEL-positive cells was assessed. Graph shows mean ±S.E.M. of quantitative analysis from six SLE patients and five healthy controls. Statistical analysis was determined by Mann–Whitney *U-*test (**P=0.0043)

An *in vitro* simulated model of cardiac H/R injury was used, whereby neonatal rat cardiomyocytes were isolated and pre-treated with 500 *μ*g/ml IgG purified from patients with SLE and HC, and then exposed to H/R injury. Pre-incubation with HC IgG resulted in an increase of 7.3% (±S.D. 3.9, *n*=5) in TUNEL-positive cells compared with untreated cells exposed to H/R injury (Figure 1). However, when cells were pre-incubated with IgG purified from patients with SLE, TUNEL positivity was significantly enhanced further to 54.2% (±S.D. 8.1, *n*=6, *P*=0.0043).

**Figure 2:**
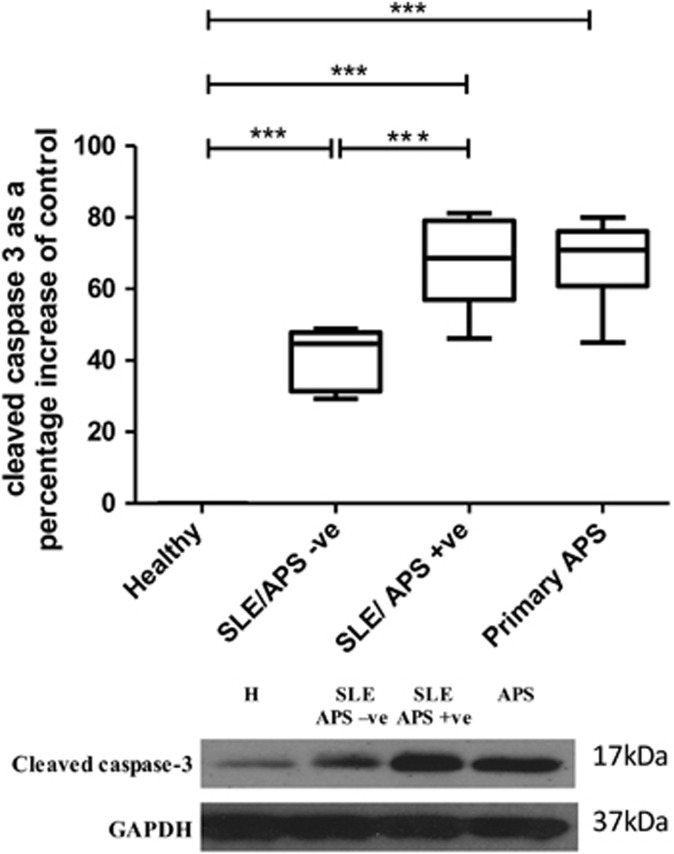
IgG from patients with SLE/APS +ve, SLE/APS –ve and APS enhances cleaved caspase-3 in neonatal rat cardiomyocytes in H/R injury. Cells were pre-treated with 500 *μ*g/ml healthy, SLE/APS negative, SLE/APS positive or primary APS IgG. The following day cells were exposed to H/R injury (4 h hypoxia+4 h reoxygenation) and the level of cleaved caspase-3 was assessed by western blot. Graph shows ±S.E.M. of quantitative analysis from five SLE/APS-positive patients, five SLE/APS-negative patients, six primary APS patients and ten healthy controls. Graphical representations of the density ratios of each protein and GAPDH expressed as a percentage of untreated cells are displayed. Statistical analysis determined by one-way ANOVA using *post hoc* tukey to compare all columns (***P<0.0005)

To assess if this pro-apoptotic role for SLE autoantibodies in this *in vitro* H/R was dependent on the presence of APS-related autoantibodies, further experiments were performed using another marker of apoptosis, cleaved caspase-3 and an expanded set of patient IgG samples allowing groups to be compared between those that are SLE/APS positive *versus* SLE/APS negative and APS alone. Figure 2 shows effects on cleaved caspase-3 upon incubation with IgG from the different subject groups. SLE-derived IgG had a pro-apoptotic effect in this model but the presence of APS-derived IgG had a significantly greater pro-apoptotic effect over and above that seen in SLE/APS-negative IgG. Compared with untreated cells, HC IgG treatment did not alter cleaved caspase-3 levels whereas these levels were significantly increased by 68.15% (±S.D. 13.5, *n*=5, *P*<0.0005) in the presence of SLE/APS IgG and by 67.94% (±S.D. 12.41, *n*=6, *P*<0.0005) in the presence of primary APS IgG but by only 40.66% (±S.D. 8.7, *n*=5, *P*<0.0005) in the presence of SLE/APS-negative IgG. Thus, since the two APS groups gave very similar results we chose to combine them in subsequent experiments reported in this paper, which all compare three groups: APS, SLE/APS negative and HC.

### The pathogenic effect of APS IgG is dependent upon enhanced phosphorylation of the pro-apoptotic MAPK p38 in neonatal rat cardiomyocytes within an *in vitro* H/R injury model

**Figure 3:**
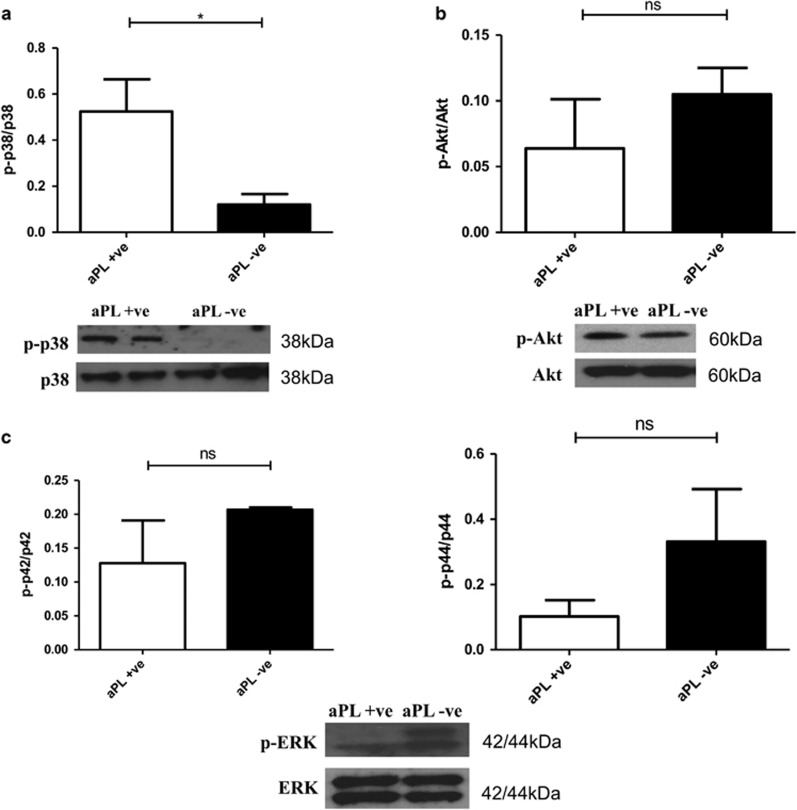
IgG purified from APS patients increases the pro-apoptotic kinase p38 MAPK phosphorylation in neonatal rat cardiomyocytes in H/R injury. Cells were pre-treated with 500 *μ*g/ml IgG (APS (primary or SLE/APS) and SLE/APS negative). The following day cells were exposed to H/R injury (4 h hypoxia+4 h reoxygenation) and the levels of p38 MAPK (**a**), Akt (**b**) and ERK 1/2 (p42/p44) (**c**) phosphorylation assessed using western blot. Graphical representations of the density ratios of each phosphorylated to total protein expressed in arbitrary scanning units are displayed. Graph shows±S.E.M. of quantitative analysis from five independent experiments. Statistical analysis determined by unpaired *t*-test (*P=0.0179)

Enhancement of MAPK p38 phosphorylation was observed in the presence of APS IgG but not SLE/APS-negative IgG when compared with cells treated with HC IgG during H/R injury (Figure 3a). The mean ratio of phosphorylated to total p38 MAPK was 0.52 (±S.D. 0.37) in cells treated with APS IgG *versus* 0.12 (±S.D. 0.12) in cells treated with SLE/APS-negative IgG (*P*=0.0174). However, APS IgG did not significantly affect levels of Akt and ERK 1/2 phosphorylation (Figures 3b and c). It should be noted that the p44 isoform of ERK 1/2 had reduced phosphorylation in the presence of APS IgG when compared with SLE/APS-negative IgG, although this was not significantly different.

**Figure 4:**
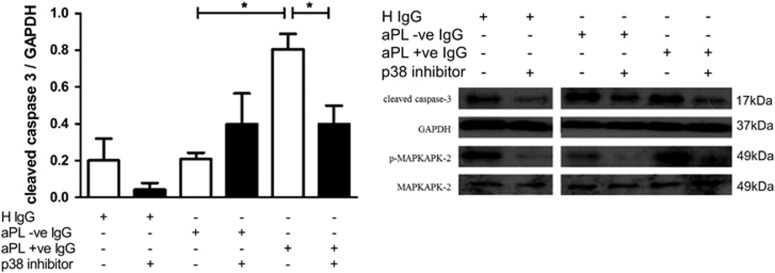
The p38 inhibitor SB23580 prevents IgG from APS patients enhancing cleaved caspase-3 in neonatal rat cardiomyocytes in H/R injury. Cells were pre-treated with 10 *μ*M SB203580 and 2 h later 500 *μ*g/ml IgG (healthy, APS positive (primary and SLE/APS) and SLE/APS negative). The following day cells were exposed to H/R injury (4 h hypoxia+2 h reoxygenation) and the levels of cleaved caspase-3 and phosphorylated MAP kinase-activated protein kinase 2 (p-MAPKAPK2) were assessed using western blot. Graph shows ±S.E.M. of quantitative analysis from four independent experiments. Graphical representations of the density ratios of each protein and GAPDH expressed in arbitrary scanning units are displayed. Statistical analysis determined by unpaired *t*-test (*P=0.0256, *P=0.0286)

Co-incubation with a p38 MAPK-specific inhibitor (SB23580) reduced the effect of APS IgG on levels of cleaved caspase-3 (Figure 4). Thus, cleaved caspase-3 levels relative to GAPDH was 0.81 (±S.D. 0.17) in the presence of APS IgG, but 0.40 (±S.D. 0.14) in the presence of APS IgG plus SB23580 (*P*=0.029). In fact, presence of this inhibitor reduced cleaved caspase-3 almost to the levels seen with SLE/APS-negative IgG −0.29 (±S.D. 0.12) but not to levels seen with HC IgG −0.12 (±S.D. 0.1). No significant effect on cleaved caspase-3 level was observed with the p38 inhibitor in cells that were untreated, or treated with HC IgG or SLE/APS-negative IgG.

### Removal of *β*_2_GPI from serum abrogates the pathogenic effect of APS-positive IgG in neonatal rat cardiomyocytes within an *in vitro* H/R injury model

**Figure 5:**
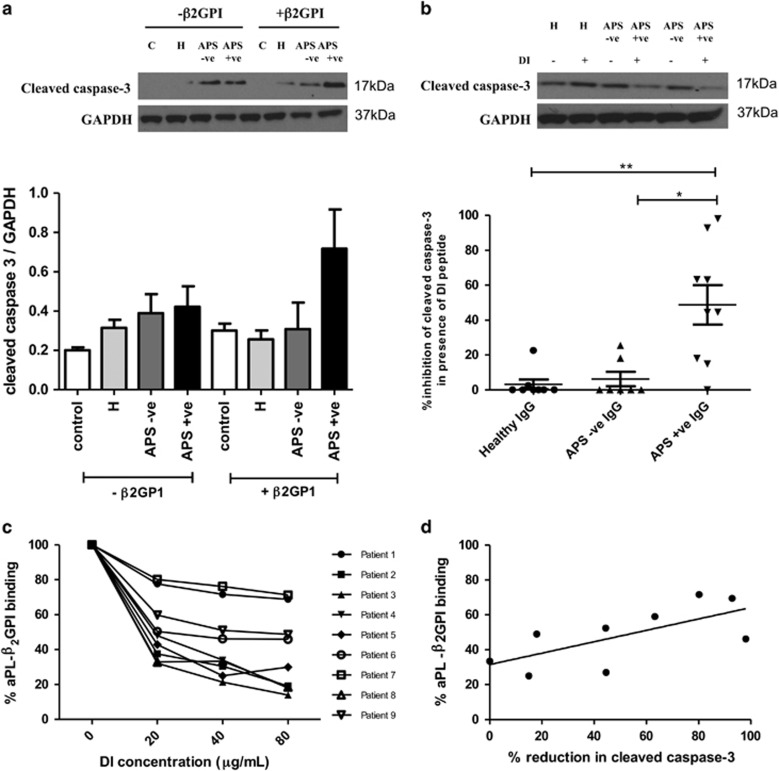
The pathogenic effect of APS-positive IgG is reduced in the presence of fluid-phase DI peptide. Neonatal rat cardiomyocytes were cultured in human *β*_2_GPI-deficient serum (+/− spiked human *β*_2_GPI at a final concentration of 200 *μ*g/ml) and pre-treated overnight with 500 *μ*g/ml IgG (pooled from 5 patients per group) (**a**). IgG purified from APS positive (primary APS or SLE/APS) (*n* = 9) SLE/APS negative (*n* = 7) or healthy controls (*n* = 8) were pre-incubated for 2 h at room temperature with 40 *μ*g of recombinant DI peptide or PBS. Cells were then treated with IgG+PBS/IgG+DI overnight (**b**). The following day cells were exposed to H/R injury (4 h hypoxia+2 h reoxygenation) and levels of cleaved caspase-3 were assessed using western blot (**a**+**b**). A competition based ELISA was performed to measure the ability of DI to bind to IgG from the APS-positive group (*n* = 9) (**c**). The percentage aPL-*β*_2_GPI binding at 40 *μ*g of DI peptide correlated with percentage reduction in cleaved caspase-3 for the APS positive (primary APS and SLE/APS positive) samples (*r*=0.6755, *P*=0.0459) (*n* = 9) (**d**). Graphical representations of the density ratios of each protein and GAPDH expressed as a percentage of increase of IgG+DI compared with IgG+PBS are displayed. Statistical analysis determined by Mann–Whitney *U-*test (**a**) (*P=0.0145, **P=0.0045)

The depletion of *β*_2_GPI from human serum led to loss of the pathogenic effect of APS IgG on cleaved caspase-3 levels when exposed to H/R injury. When cells cultured in *β*_2_GPI-deficient serum were spiked with human *β*_2_GPI, the pathogenic effect of APS-positive IgG was restored (Figure 5a).

### A recombinant human DI decoy peptide blocks the pathogenic effect of APS-positive IgG in neonatal rat cardiomyocytes within an *in vitro* H/R injury model

Purified IgG was pre-incubated with recombinant DI for 2 h prior to incubation with cells. In the presence of the DI peptide the effect of APS IgG on cleaved caspase-3 was inhibited (Figure 5b). In cells treated with APS IgG and exposed to H/R injury, the addition of DI peptide led to a 48.81% (±S.D. 34.07) inhibition in cleaved caspase-3 level. Addition of DI caused much smaller reductions in levels of cleaved caspase-3 in cells treated with HC IgG (reduction 3.14% (±S.D. 7.9)−*P*=0.0045 compared with APS IgG) or SLE/APS-negative IgG (reduction 6.2% (±S.D. 10.84)−*P*=0.0145 compared with APS IgG).

In addition, the ability of the recombinant DI to inhibit binding of IgG from the APS patient cohort to solid-phase *β*_2_GPI was measured by ELISA. Importantly, serum from all nine patients had demonstrated high activity in our in-house solid-phase *β*_2_GPI ELISA assay. The ability of recombinant DI at concentrations ranging from 0 to 80 *μ*g/ml to inhibit binding of all nine sera to solid-phase β_2_GPI in this assay was then tested. Figure 5c shows that six serum samples were inhibited by the presence of the DI peptide to different degrees. Figure 5d compares the reduction in cleaved caspase-3 caused by DI in cultured cardiomyocytes exposed to APS IgG samples from individual patients with the percentage by which binding to *β*_2_GPI of the same APS IgG samples is inhibited by DI at the same concentration (40 *μ*g/ml). There was a positive correlation between these two separate measures of DI inhibition of these samples (*r*=0.6775, *P*=0.0459).

## Discussion

A novel pathogenic role for aPL autoantibodies has been identified by demonstrating a pro-apoptotic effect within an *in vitro* model of cardiomyocyte H/R injury. Furthermore, the mechanism through which this pathogenic effect may be mediated has been dissected using a range of time points to fully understand this complex interplay of signalling pathways. Fleming *et al.*^13^ have previously shown that in a mesenteric I/R injury model that anti-*β*_2_GPI autoantibodies restore local and remote tissue damage in a Complement Receptor 2/Complement Receptor 1-Deficient mouse model that is typically protected from I/R injury. Owing to previous studies showing that I/R injury mechanisms are similar in a variety of tissue types^13^ and patients with APS having a greater risk of myocardial infarction,^24^ one may hypothesise that aPL antibodies play a pathogenic role also in the setting of the myocardium. This study has found that IgG purified from patients with SLE and/or APS enhanced apoptosis, as measured by an increase in TUNEL positivity (Figure 1). Levels of cleaved caspase-3 were also assessed in a larger cohort of patients and this pathogenic effect was confirmed (Figure 2). Moreover, enhanced cleaved caspase-3 was most pronounced in those patients who were APS positive (SLE/aPL positive and primary APS), suggesting an aPL-dependent mechanism of pathogenicity distinct from patients with SLE.

Experiments were performed to identify potential mechanisms through which IgG from patients with APS caused pathogenicity in this H/R injury model. We found that APS IgG enhanced p38 MAPK phosphorylation significantly more than SLE/APS negative or HC IgG and that a p38 MAPK inhibitor (SB203580) reduced the increase in cleaved caspase-3 caused by APS IgG (Figure 4) This suggests that the enhanced pro-apoptotic effect of APS IgG is due to increased phosphorylation of the kinase p38 MAPK. The use of p38 MAPK inhibitors in the treatment of cardiac I/R injury is currently being explored in a number of trials.^25^ In untreated cells, while p38 MAPK is enhanced by H/R injury, levels observed are low compared with APS IgG-treated cells. It could therefore be suggested that the p38 MAPK contribution to H/R injury in the presence of a p38 MAPK stimulus such as APS IgG is greater and therefore an inhibitor would be more effective in reducing the pathogenic effects mediated by p38 MAPK in patients with APS.

It is acknowledged that in APS the dominant population of pathogenic autoantibodies target the circulating autoantigen *β*_2_GPI.^26, 27, 28, 29^ To demonstrate if this APS IgG-mediated pathogenicity of cardiomyocyte H/R injury was mediated through *β*_2_GPI autoantibodies, cells were cultured in media containing *β*_2_GPI-deficient human serum in the presence of IgG from the different groups studied. It was observed that in the absence of this serum co-factor, there was a reduction in cleaved caspase-3 in the presence of APS IgG (Figure 5a), but when human *β*_2_GPI was added back into the system to a final concentration typically observed in humans, levels of cleaved caspase-3 were enhanced back to levels similar to that seen previously. This result did not reach statistical significance, with a noted limitation of this experiment being that pooled IgG was used, therefore potentially dampening the response observed by specific patient samples.

Although SLE/APS-negative IgG samples did have an effect on cleaved caspase-3 it was smaller than that of APS IgG. Also SLE/APS-negative IgG had no effect on MAPK p38 phosphorylation. This suggests an alternative mechanism of pathogenicity for this polyclonal population of IgG. Many different autoantibody specificities have been identified in SLE. Anti-Ro antibodies can affect the fetal heart causing neonatal lupus play and could also have effects in adults;^30^ however, clinical data confirm that the majority of our patients are anti-Ro negative. It is possible that a yet to be identified population of antibodies may be pathogenic in this model of cardiomyocyte H/R injury. Further studies are now required to try and identify both the autoantibody populations and the epitopes to which they bind to cause a pathogenic effect.

Studies using domain deletion mutants of *β*_2_GPI, in which one or more domains are missing, have shown that the immunodominant epitope which binds the pathogenic anti-*β*_2_GPI APS autoantibodies is located in DI^5, 6^ and recombinant DI can bind these APS autoantibodies in both the solid and fluid phase.^31, 32^ Pre-incubating APS-positive IgG from individual patients with recombinant human DI, prior to treatment of cells, reduced cleaved caspase-3 in H/R injury (Figure 5b). This suggests that DI inhibits the pathogenic effect of APS-positive IgG through a decoy mechanism, in which it binds the APS during the pre-incubation stage, therefore causing a protective effect. Additionally, the serum from the APS-positive IgG samples selected were tested in a competition based ELISA, to see if fluid-phase recombinant DI could inhibit IgG from binding to solid-phase *β*_2_GPI. It was observed that increasing DI concentrations reduced binding (Figure 5c), which correlated with reductions in cleaved caspase-3 (Figure 5d), further supporting the case that it is anti-DI antibodies that are the dominant pathogenic population in this *in vitro* H/R cardiac injury simulation model. However, to definitively prove that anti-DI are the pathogenic subpopulation of aPL that enhance cardiomyocyte H/R injury, these antibodies must be affinity purified from multiple patients and then tested in an *in vivo* cardiac H/R model, ideally compared with affinity purified anti-*β*_2_GPI antibodies binding to domains other than DI. The results presented in this article underpin these detailed future *in vivo* studies.

The next question to be answered is what cell surface receptors *β*_2_GPI is binding to, to induce this response. It has previously been shown that *β*_2_GPI has the ability to bind to a range of receptors including TLR4, TLR2, Annexin A2 and Ro 60.^33, 34, 35, 36^ A known downstream activator of TLR4 is p38 MAPK^22^ and therefore it could be suggested that the APS autoantibodies are mediating their pathogenic effect via this receptor. Additionally, TLR4 has been implicated in I/R injury, with TLR4-deficient mice showing cardioprotection in an *in vivo* I/R injury model.^37, 38^ Further experiments are now required to confirm this.

In humans, there is no large-scale study to our knowledge that has compared cardiac infarct sizes between patients with APS±SLE and age- and gender-matched controls. This *in vitro* study provides the first evidence that autoantibodies, a prominent feature of patients with APS±SLE, may play a role in the degree of damage to the myocardium in I/R injury and propose a novel non-thrombotic role for these autoantibodies in this organ. Further studies using affinity purified anti-DI antibodies within a passive transfer animal model of cardiac I/R injury to confirm pathogenicity *in vivo* are now required. The outcome of this research may identify a new mechanism through which antibodies from patients with APS±SLE may cause cardiac damage following an MI. This research could then underpin future clinical studies to determine if patients with APS±SLE are susceptible to a larger MI and/or stroke as compared with age- and gender-matched controls. Ultimately, this may lead to identification of novel pathogenic roles for antibodies from patients with APS±SLE and investigation of potential new targets and therapeutics with the aim of ultimately improving outcomes for patients with these conditions and cardiovascular disease.

## Materials and Methods

### Patients

Serum samples were obtained from 35 individuals for this study with informed consent and local ethical approval in accordance with the Declaration of Helsinki. We studied three groups of subjects. The first group of 11 patients had APS; 5 with SLE-associated APS (1 vascular thrombosis (VT) and pregnancy morbidity (PM) and 4 VT) and 6 with primary APS (1 PM and 5 VT). The second group of nine patients had SLE but no serum aPL and no clinical features of APS. The third group were 15 healthy controls (HCs) who also had no serum aPL. All patients with APS and SLE fulfilled the current accepted classification criteria.^39, 40^

### Immunological characterisation and purification of IgG

IgG was protein G purified, passed through endotoxin removal columns (Pierce, Thermo-Fisher, Basingstoke, UK), and confirmed to be <0.06 endotoxin units per millilitre by Limulus ameboycte lysate assay (Sigma-Aldrich, St. Louis, MO, USA) as described previously.^41^ Total IgG concentration was determined by spectrophotometry. Serum and IgG aCL, anti-*β*_2_GPI and anti-DI titres were determined by enzyme-linked immunosorbent assay (ELISA) as described previously.^31^ Serum levels are reported in Table 1, activity levels were re-confirmed after IgG purification and activity was retained (data not shown).

### Expression and purification of recombinant DI

Expression of wild-type recombinant human DI in *Escherichia coli* and purification using cytoplasmic inclusion bodies from which DI can be re-folded was performed as described previously.^42^ Subsequent cleavage with factor Xa removed the hexahistidine tag, resulting in native DI peptide.

### Competitive inhibition assay

A competitive inhibition ELISA was used to assess the ability of recombinant DI in the fluid phase to inhibit binding of aPL to solid phase whole *β*_2_GPI. Prior to the assay, our in-house standard *β*_2_GPI ELISA previously described^32^ was used to assess at what dilution of serum corresponded to 50% of the maximal binding absorbance unit of the serum. This ELISA was then repeated with the following amendment – anti-*β*_2_GPI inhibition curves were constructed by pre-incubating sera from patients with APS with varying concentrations of recombinant DI (ranging from 0 to 80 *μ*g/ml) for 2 h at room temperature prior to addition to a 96-well plate.

### Primary neonatal rat ventricular cardiomyocyte isolation

Primary cardiomyocytes were isolated as previously described.^43^ All animal studies were approved by the University College London Biological Services Ethical Review Committee and licensed under the UK Home Office regulations and the Guidance for the Operation of Animals (Scientific Procedures) Act 1986 (Home Office, London, UK). All isolation buffers were oxygenated by bubbling medical grade oxygen through the solution for 5 min prior to use. Neonate rat pups (Sprague-Dawley) that were <2day postpartum were decapitated and rinsed in ethanol. The hearts were removed by cutting along the sternum and dissecting the heart through the chest wall. Hearts were washed in isolation buffer (116 mM NaCl, 20 mM HEPES, 0.77 mM NaH_2_PO, 5.5 mM glucose, 5.4 mM KCl, 0.4 mM MgSO containing 600 *μ*g/ml collagenase type II and 250 μg/ml pancreatin) and cut into small 2 mm pieces and then incubated at 37 **°**C until plating. The digestion procedure was repeated seven times and the pellets pooled and pre-plated for 1 h in 15% (v/v) fetal bovine serum (FBS) in DMEM to remove contaminating fibroblasts. Cardiomyocytes were then seeded at 2 × 10^6^ cells per well of a six-well plate, which had been pre-coated with 1% (w/v) gelatin (Sigma-Aldrich). The DMEM containing 15% (v/v) FBS was replaced the following day with maintenance media (DMEM containing 1% (v/v) FBS) or 1% Human Apo H Deficient Serum (Assay Pro). For IgG incubations cells were pre-treated with 500 *μ*g/ml purified polyclonal IgG from patient or healthy control serum overnight prior to H/R injury. For DI peptide experiments, IgG was pre-incubated with 40 *μ*g recombinant DI peptide for 2 h at room temperature, prior to incubation with cells. A control experiment was performed, whereby cells were incubated with bovine serum albumin (BSA) at 500 *μ*g/ml to confirm that the effect observed was IgG specific. No difference in cleaved caspase-3 was observed when compared with untreated cells (data not shown).

### *In vitro* H/R injury

Cell culture media was replaced with ischaemic buffer (137 mM NaCl, 12 mM KCl, 0.49 mM MgCl, 0.9 mM CaCl_2_.H_2_0, 4 mM HEPES, 20 mM sodium lactate and 10 mM deoxyglucose (pH 6.2)) and cells transferred to an in-house built anoxic chamber pre-warmed to 37 °C. Simulated ischaemia was achieved by addition of 5% CO_2_ in balanced argon to exclude any oxygen. Cells were subjected to simulated ischaemic injury for 4 h after which ischaemic buffer was replaced with DMEM containing 1% (v/v) FBS and cultured in 5% CO_2_ in air (simulated reperfusion) for the indicated times. Optimisation of H/R injury was performed using TUNEL (Supplementary Figure S1).

### Determination of apoptosis

Cells were seeded on UV-irradiated coverslips and fixed in 4% (w/v) paraformaldehyde in phosphate-buffered saline (PBS) for 15 min at room temperature. *In situ* cell death was detected in cultured cardiomyocytes by using TUNEL assay kit (Roche Diagnostics, Meylan, France) according to the manufacturer's instructions. Cells were then washed and cell nuclei counter stained with DAPI. Coverslips were mounted onto glass slides using a Zeiss Axioscope inverted fluorescence microscope (Zeiss, Oberkochen, Germany).

### Western blotting

Cells were lysed in 1 × RIPA buffer (20 mM TRIS-HCl (pH 7.5), 150 mM NaCl, 1 mM EDTA, 1 mM EGTA, 1% (v/v) IGEPAL, 1% (w/v) deoxycholate, 250 mM sodium pyrophosphate and Complete-mini protease inhibitor cocktail (Roche, Basel, Switzerland) and incubated on ice for 15 min. Lysates were spun at 13 000 r.p.m. for 5 min to pellet cell debris. The supernatant was transferred to a clean tube and assayed for protein using the BCA assay kit according to the manufacturer's instructions (Pierce). Twenty micrograms total protein was run on denaturing PAGE gels in protein running buffer (25 mM TRIS-HCl, 192 mM glycine, 0.1% (w/v) sodium dodecyl sulphate) and wet-transferred in transfer buffer (25 mM TRIS-HCl, 192 mM glycine, 0.1% (w/v) sodium dodecyl sulphate containing 20% (v/v) methanol) to Hybond-C nitrocellulose membrane (GE Healthcare, Little Chalfont, UK). Membranes were blocked for 60 min in 5% (w/v) non-fat dry milk in PBS for 1 h at room temperature before being incubated with primary antibody (Cell Signalling Danvers, MA, USA) overnight at 1/1000 dilution in 5% (w/v) non-fat dry milk in PBS at 4 **°**C. The following day they were washed three times in PBS containing 0.1% (v/v) Tween-20. Horseradish-peroxidase conjugated secondary antibodies (DAKO, Ely, UK) were used at 1/5000 dilution in 5% (w/v) non-fat dry milk for 1 h at room temperature and then washed three times in PBS containing 0.1% (v/v) Tween-20. Bands were visualised using enhanced chemiluminescence (GE Healthcare, UK).

### Statistical analysis

Statistical analyses were performed using GraphPad Prism software 4.0c (GraphPad Software, La Jolla, CA, USA). Normally distributed data were analysed by parametric *t*-test and non-normally distributed data by non-parametric Mann–Whitney *U-*test. For multiple means comparisons, one-way analysis of variance (ANOVA) followed by Tukey *post hoc* test was used to determine statistical significance.

### Study approval

All animal studies were approved by the University College London Biological Services Ethical Review Committee and licensed under the UK Home Office regulations and the Guidance for the Operation of Animals (Scientific Procedures) Act 1986 (Home Office, London, UK).

## Figures and Tables

**Table 1:** Clinical characteristics and binding properties of patient cohort

	**Primary APS (*n*=6)**	**SLE/aPL +ve (*n*=5)**	**SLE/aPL –ve (*n*=9)**	**Healthy controls (*n*=15)**
Age (mean±SEM)	55 (±4.5)	45.2 (±5.1)	42.8 (±6.3)	35.2 (±4.7)
Sex	3 F/3 M	5 F	8 F	8 F/2 M
Serum aCL (mean GPLU±SEM)	50.0 (±20.65)	43.26 (±15.83)	3.62 (±0.34)	4.12 (±0.58)
Serum anti-*β*_2_GPI (mean SU±SEM)	91.6 (±19.7)	49.62 (±16.1)	3.58 (±0.3)	5.4 (±0.7)
Serum anti-DI (mean as a % binding to an in-house standard)	35.56 (±38.96)	11.85 (±3.98)	4.20 (±2.57)	3.56 (±1.78)

Patients with APS (*n*=11) are separated into clinical groups (primary APS and SLE/APS positive). The mean age (±S.D.) of each patient subgroup and for the HC group is also shown. Purified polyclonal IgG was tested in aCL, anti-*β*_2_GPI and anti-DI (of *β*_2_GPI) direct binding assays and mean values (from duplicate experiments±S.D.) are shown here. For aCL, mean activity is shown in GPLU, anti-*β*_2_GPI is shown in GDU (IgG arbitrary units) and anti-DI as the percentage activity of an in-house standard

## Abbreviations

MAPK: mitogen-activated protein kinase
MI: myocardial infarction
I/R: ishaemia/reperfusion
aPL: antiphospholipid
DI: domain I
B_2_GPI: beta-2 glycoprotein I
APS: antiphospholipid syndrome
SLE: systemic lupus erythematosus
TUNEL: terminal deoxynucleotidyl transferase dUTP nick-end labelling
CL: cardiolipin
H/R: hypoxia/reoxygenation
HCs: healthy controls
VT: vascular thrombosis
PM: pregnancy morbidity
