# Supplementary figures and images for: Antiphospholipid antibodies enhance rat neonatal cardiomyocyte apoptosis in an in vitro hypoxia/reoxygenation injury model via p38 MAPK
Source: Cell Death Dis. 2017 Jan 12;8(1):e2549–. doi: 10.1038/cddis.2016.235 (PMC5386347; doi:10.1038/cddis.2016.235)

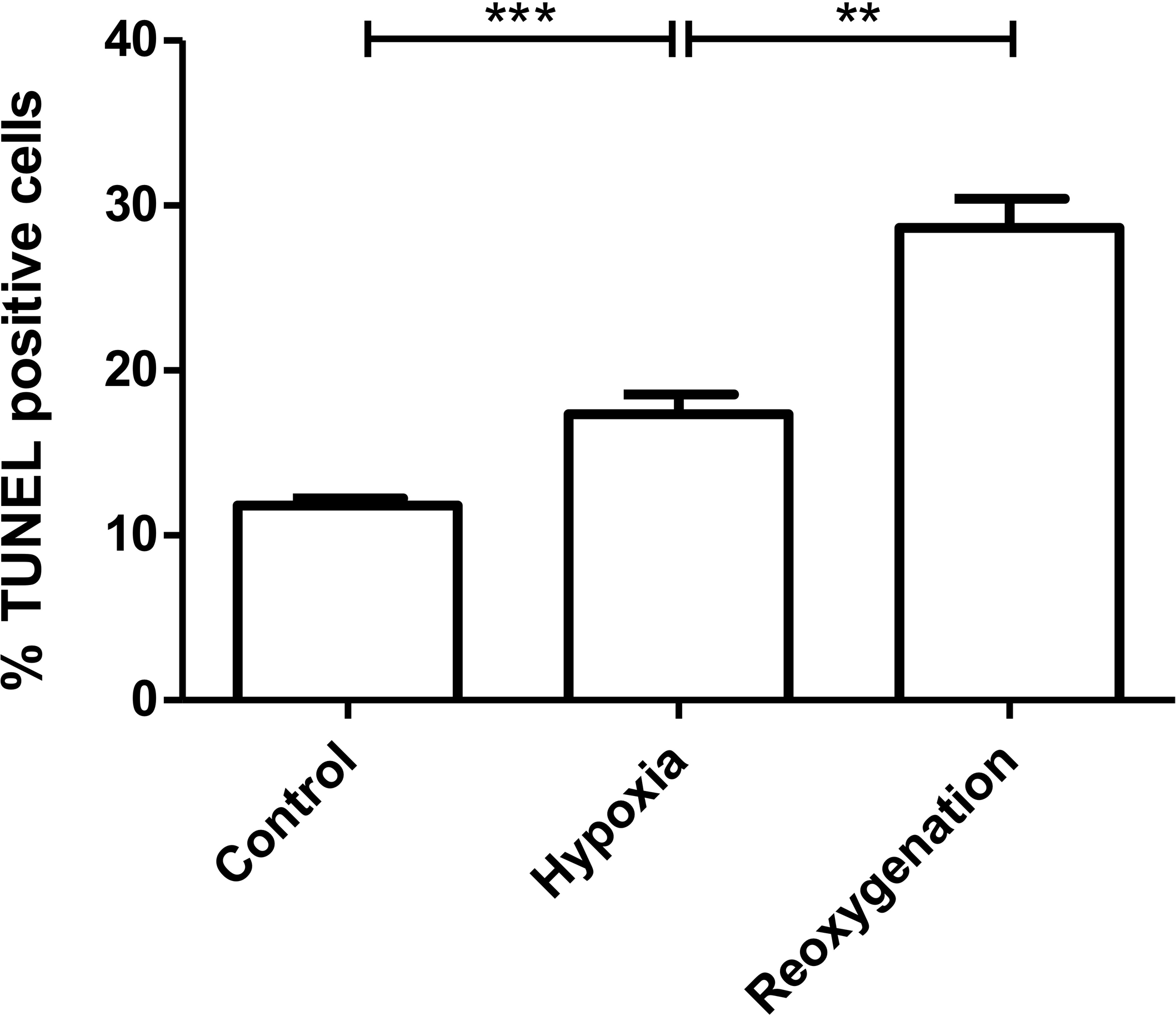

Supplement: Supplementary Figure 1 [file cddis2016235x1.tif]
