# Supplementary material for: Antiphospholipid antibodies enhance rat neonatal cardiomyocyte apoptosis in an in vitro hypoxia/reoxygenation injury model via p38 MAPK
Source: Cell Death Dis. 2017 Jan 12;8(1):e2549–. doi: 10.1038/cddis.2016.235 (PMC5386347; doi:10.1038/cddis.2016.235)
Supplement: Supplementary Figure Legend [file cddis2016235x2.docx]

**Supplementary Figure 1. Optimisation of a in vitro model of H/R injury.** Neonatal rat cardiomyocytes were exposed to simulated ischemia (hypoxia) for 4 hours and/or simulated reperfusion 16 hours (reoxygenation). Cells were fixed in 4% PFA and the percentage of TUNEL positive cells was assessed. Graph shows mean ±SEM of quantitative analysis from four independent experiments. Statistical analysis determined by 1 way ANOVA using post-hoc tukey to compare all columns (**p<0.005, *** p<0.0005).
